# Successful endoscopic sphincterotomy using a rotatable sphincterotome in a patient with situs inversus totalis

**DOI:** 10.1055/a-2817-4568

**Published:** 2026-03-16

**Authors:** Kenshin Tamura, Yuki Kawasaki, Hisaki Kato, Kazuya Sumi, Jun Ushio, Noboru Yokoyama, Haruhiro Inoue

**Affiliations:** 1 378609 Digestive Diseases Center, Showa Medical University Koto Toyosu Hospital, Koto, Japan

**Fig. 1:**
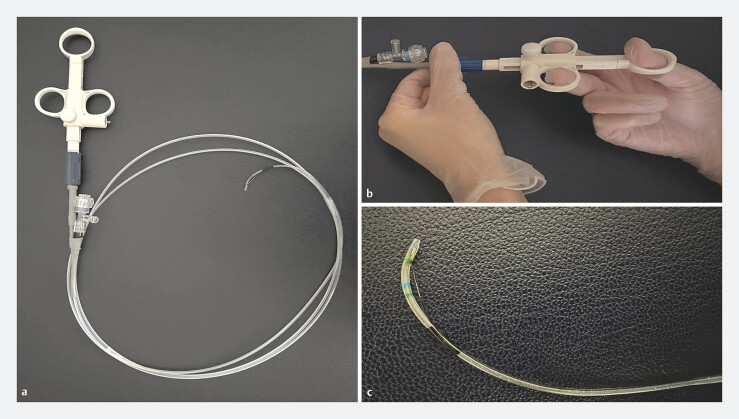
The rotatable sphincterotome. **a** Device overview. **b** Rotatable handle. **c** Blade at the catheter tip.

Situs inversus totalis (SIT) is a rare congenital condition characterized by mirror-image transposition of the thoracic and abdominal organs 1 . In patients with SIT, endoscopic sphincterotomy (EST) is technically challenging because the optimal incision direction differs from that in normal anatomy. Although several reports have described endoscopic retrograde cholangiopancreatography (ERCP) or EST in SIT cases, most have focused on feasibility and safety, and only a limited number have detailed active adjustment of the sphincterotome under mirror-image anatomy 2 3 . Recently, a novel rotatable sphincterotome has become available ( Fig. 1 **a** , Fig. 1 **b** , Fig. 1 **c** ) and its usefulness in patients with surgically altered gastrointestinal anatomy has been reported 4 . This sphincterotome can rotate over a wider range than conventional rotatable sphincterotomes, enabling more flexible and precise adjustment of the cutting direction.

**Fig. 2:**
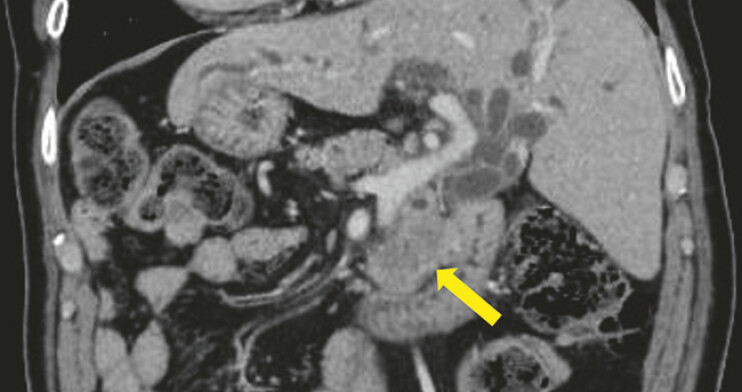
Computed tomography (CT) demonstrating situs inversus totalis (SIT) and pancreatic cancer (yellow arrow).

**Fig. 3:**
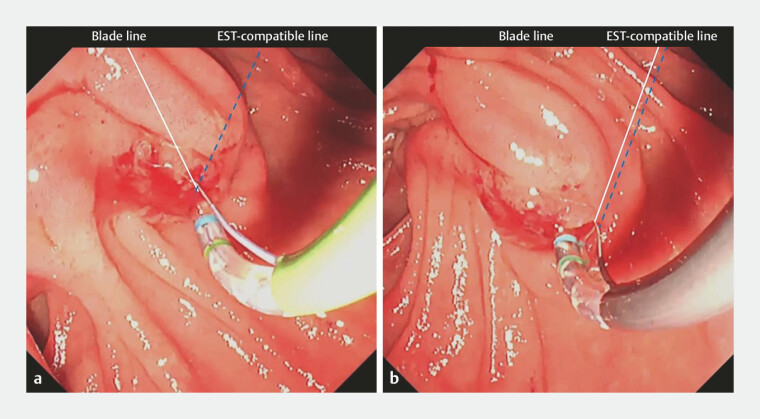
Endoscopic images during ERCP. **a** The blade of the conventional sphincterotome was initially facing in the 11 o’clock direction. **b** The blade of the novel rotatable sphincterotome was rotated in the 1 o’clock direction by turning the handle.

**Fig. 4:**
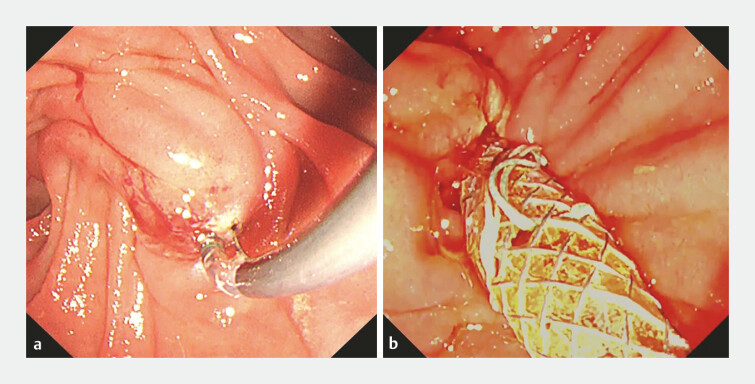
**a** Endoscopic sphincterotomy (EST) completed using the rotatable sphincterotome. **b** A biliary metallic stent was placed after EST.

A man in his 70s with SIT presented with jaundice. Computed tomography (CT)

demonstrated SIT and pancreatic head cancer ( Fig. 2 ). ERCP was performed for biliary drainage using the 360-degree turn technique, which does not require repositioning of either the patient or endoscopic system. After reaching the papilla and achieving selective biliary cannulation, EST was attempted with a conventional sphincterotome; however, the blade could not be oriented in the appropriate direction ( Fig. 3 **a** ). Therefore, we switched to the novel rotatable sphincterotome. After clockwise extracorporeal rotation, the blade was correctly aligned under endoscopic visualization, allowing successful EST in the 1 o’clock direction ( Fig. 3 **b** ). Notably, correct blade orientation was achieved solely by extracorporeal rotation, without the need for further adjustment under endoscopic visualization. A biliary metallic stent was subsequently placed ( Fig. 4 **a** , Fig. 4 **b** ), leading to improvement in jaundice with no ERCP-related adverse events ( Video 1 ).

Several approaches to ERCP in patients with SIT have been reported 5 . In this case, the 360-degree turn technique allowed ERCP to be performed without repositioning either the patient or the endoscopic system. Because endoscopic images are mirror-reversed in SIT, precise adjustment of the incision direction is challenging. This novel rotatable sphincterotome enables safe and effective EST during the 360-degree turn technique in patients with SIT.

Endoscopic sphincterotomy (EST) using a rotatable sphincterotome in a patient with situs inversus totalis (SIT).Video 1
